# COVID-19 Infection Prevention Preparedness, Practices and Case Management in the Primary Health Care Units in Ethiopia

**DOI:** 10.4314/ejhs.v33i2.6S

**Published:** 2023-10

**Authors:** Habtamu Sime Gizaw, Biru Abdissa Mizana, Muluemebet Abera, Mohammed Mecha, Meskerem Jisso, Netsanet Abera, Akalewold Alemayehu, Anteneh Gadisa, Rekiku Fikre, Binyam Tilahun, Berhanu Fikadie Endehabtu, Tajebew Zayede Gonete, Kassahun Dessie Gashu, Dessie Abebew Angaw, Alemu Tamiso, Abdurezak Umer, Mesfin Kebede, Hussen Mohammed, Bekele Yazie, Kassu Ketema Gurmu, Elias Ali Yesuf

**Affiliations:** 1 Jimma University, Institute of Health, Jimma, Ethiopia; 2 Hawassa University, College of Medicine and Health Sciences, Ethiopia; 3 University of Gonder, College of Medicine and Health Science, Institute of Public Health, Ethiopia; 4 eHealthLab Ethiopia, University of Gondar, Ethiopia; 5 Dire Dawa University, College of Medicine and Health Sciences, Ethiopia; 6 World Health Organization Country Office for Ethiopia, Universal Health Coverage/Life Course, Health System Strengthening Team, Addis Ababa, Ethiopi

**Keywords:** COVID-19, Infection prevention and control, Preparedness and case management

## Abstract

**Background:**

COVID-19 as pandemic declared by WHO on March 11, 2020 and first case detected in Ethiopia on March 13/2020. The COVID-19 caused a global crisis, including millions of lives lost, public health systems in shock and economic and social disruption. Strategies depend on how an existing health system is organized. Even though public health emergency operation centers of the Ethiopia switched to emergency response, there is no national evidence about infection prevention and control. Therefore, this project aimed to assess the level of infection prevention and control and management of COVID- 19 in Ethiopia, 2021.

**Methods:**

The cross-sectional study conducted at four regions and one city (Amhara, Oromia, SNNPR, Sidama Region, and Dire Dawa). Being with zonal health departments and woredas health offices, primary health care units were selected. The data were collected electronically through Kobocollect software from November 08-28/2021. Descriptive analysis like frequency and percentage was conducted by SPSS software version 25 and the results were presented by tables, figures and narration.

**Results:**

Data were collected from 16 hospitals, 92 health centers, and 344 health posts. All hospitals have designated COVID-19 focal person. There were significant number of woredas and PHCUs who didn't have IPC guidelines and protocols. About 11 woredas had no any type of diagnostic tests for COVID-19.

**Conclusions:**

The study revealed that there were significant gaps on Infection prevention and control practice, shortage of personal protective equipment, isolation and specimen transportation problem, lack of call centers. We recommend concerned bodies to fill the identified gaps.

## Introduction

The World Health Organization (WHO) has declared COVID-19 as pandemic on March 11, 2020(1). In Ethiopia, few days following the declaration of the pandemic the first COVID-19 case was confirmed on March 13/2020. Resources and staff are being diverted to test and provide treatment for people with presumed or diagnosed COVID-19, and supplies are limited. The problems get worse in developing countries where resources are limited and with shortages of trained health care providers. Health services are being compromised in order to meet the demands of caring for COVID-19 patients (2-4).

The COVID-19 has caused an unprecedented global crisis, including millions of lives lost, public health systems in shock and economic and social disruption, disproportionately affecting the most vulnerable peoples. According to the daily report of WHO, globally, as of the end of May, 2022, there are over 531 million confirmed cases and over six million COVID-19 deaths, and in Ethiopia there were about 472 thousand confirmed cases and 7,513 deaths (5). The pandemic has challenged local, national, and global capacities to prepare and respond. The various national strategies taken to control the pandemic are widely debated (6). However, the relative success of these strategies depends largely on how an existing health system is organized, governed and financed across all levels in a coordinated manner. The pandemic has exposed the limitations of many health systems, including some that have been previously classified as high performing and resilient (7).

In Ethiopia, following the confirmation of the first COVID-19 case in the country on 13^th^ March 2020, the national and regional health workforce focused to emergency response mode. To accelerate the pace of the response in an intensified and decentralized manner, strategic approaches have been adopted to proactively respond to the pandemic meanwhile continuity of essential health services might have been affected (8, 9). Decline in essential health services is attributed to several factors, such as shifting of human resources from essential health services to COVID-19 response, fear of infection due to lack of Personal Protective Equipment (PPE), increased workload due to exposure of health care providers and lack of readiness among health care providers to continue health care services during the pandemic (10).

Public health emergency operation centers of the Ethiopia switched to emergency response mode and some health facilities has been reassigned as COVID-19 treatment center. When health systems are overwhelmed, countries need to make problematic decisions to balance the demands of responding to COVID-19, while concurrently to maintain essential health service delivery. The aim of this research project is to assess level of covid-19 infection prevention preparedness, practices and its implantations in the primary health care unit facilities in Ethiopia.

## Materials and Methods

**Study Design and Setting:** The cross-sectional study conducted at the national level in four regions (Amhara, Oromia, Southern Nation Nationalities of People (SNNP) and Sidama Region) and one city administration (Dire Dawa). The data were collected from November 08-28/2021.

**Population and Sampling:** The selections of study site were done with respective zonal health department and woreda health offices. 453 primary health care units (16 primary hospitals, 92 health centers and 344 health posts) were selected from four regions and one city administration.

**Data collection procedure and Data Quality Control:** The standard tools designed by WHO for this purpose was used to collect data. The tools included the IPC measures at PHCUs, availability of PPE, and management of suspected nad confirmed COVID-19 cases in the PHCUs. Data collectors and supervisors were trained for four days. Pre-test was done before departing for the actual data collection and some adjustments were made on the tools. The KoboCollect form was prepared with strict rules to assure consistency of entry across the questions and legal value entries for responses. Data were collected by 54 health professional data collectors and 17 supervisors by Electronic KoboCollect data collection tool through face-to-face interview of the primary health care unit directors and/or others delegated. Close follow up by the research team and supervisors was undertaken. The completed forms were downloaded on daily bases to monitor the progress as well as the completeness of the form and feedbacks were also given on the daily base before the next date of data collection.

**Data Management and Analysis:** The data were exported from the Kobocollect to Excel then to SPSS version 25 for analysis. A descriptive analysis like frequency, percentage and cross tabulation conducted to describe the PHCUs IPC status, PPE availability and COVID-19 case management status.

**Ethics approval and consent to participate in data:** Ethical clearance was obtained from the Institutional Review Board of the University of Gondar, Jimma University, Hawassa University and Dire-Dawa University. Official permission letter was written from the Regional Health Bureau to Zonal health departments, Woreda Health offices and health facilities. Permission to conduct the study in each organization was asked through a letter. Informed consent was obtained from all study respondents and participants after adequate information about the study had been provided. The collected data were treated and kept confidentially.

## Results

**Figure 1:**
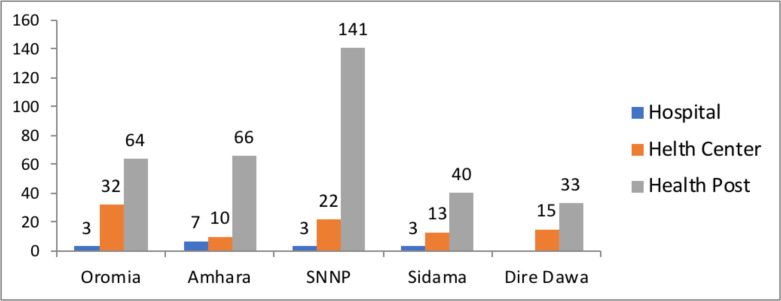
Primary Health Care Units distribution, Ethiopia, November, 2021

**Organizational characteristics:** Sixteen hospitals, ninety-two health centers and three hundred and forty-four health posts were included from four regions and one city administration (Fig. 1).

**Service interruption at PHCU and districts in the last year:** From the assessment, 13 (30.2%) of woreda health offices, 20 (21.7%) of HCs and 10 (55.6%) hospitals have reported there was a service interruption in the last year. Service interruption was the highest at primary hospitals than other PHCU.

**Common reasons for service interruption at PHCUs and districts:** Shortage of medical equipment (42%), human resources (24%), finance (15.2%) and infection prevention and patient safety supplies (18.8%) were the major reasons for service interruption at PHCUs and districts.

**Diagnosis and treatment of communicable diseases at health posts:** The assessment showed that only 93 (23%) health posts had delivered tuberculosis treatment services at full scale, and 142(41.3%) did not deliver TB treatment services. Malaria diagnosis and treatment services is available fully at 226(65.7%) of health posts, whereas 66 (19.2%) of HPs did not deliver malaria diagnosis and treatment services.

The commonest reasons for service unavailability were shortage of drugs (anti TB and malaria), medical equipment (vital sign equipment) and supplies, skill gaps, and some think it is not their scope.

**Diagnosis and treatment of communicable and NCD at health centers:** In the assessed health facilities, HIV/AIDS diagnosis and treatment (43.5%), cervical cancer screening & treatment (33.7%), cardiovascular disease (27.2%), and mental health problem diagnosis and treatment (14%) were the least available essential health services at HCs, whereas malaria (91.3%), STI (91.3), and TB (88%) diagnosis and treatment were the most available services at HCs (Table 1).

**Table 1:** Infection prevention and control (IPC) measures by hospitals in Ethiopia, 2021

Measures	Hospital (n = 16)		Health Center (n=92)	
	
	Yes (%)	No (%)	Yes (%)	No (%)
Any measures to create a COVID-19 safe environment	16(100)	-	89(96.7)	3(3.2)
Temperature screening at a dedicated entrance	12(75)	4(25)	74(80.4)	18(19.6)
Distancing of at least 2 meter	11(68.8)	5(31.2)	79(85.9)	13(14.1)
Displaying instructions on hand and respiratory hygiene practices for patients and visitors	13(81.3)	3(18.7)	86(93.5)	6(9.5)
Screening and triage of patients for suspected COVID-19	12(75)	4(25)	67(72.8)	25(27.2)
COVID-19 isolation areas clearly identified and divided from non-COVID-19 areas	14(87.5)	2(12.5)	54(58.7)	38(41.3)
Designated staff entrance for screening	10(62.5)	6(37.5)	50(54.3)	42(45.7)
Hand hygiene stations at all points of care	14(87.5)	2(12.5)	79(85.9)	13(14.1)
Use of PPE by staff	12(75)	4(25)	85(92.4)	7(7.6)
Requiring patients to wear face mask	12(75)	4(25)	66(71.7)	26(28.3)
Environment cleaning and disinfection	11(68.8)	5(31.2)	85(92.4)	7(7.6)

**Infection Prevention and control (IPC) measures at Health Posts in Ethiopia:** More than two third of health posts were created measures to create a COVID-19 safe environment, prepared hand hygiene stations, displayed instructions on hand, prevention and control measures of COVID-19 infections. Nearly halves of health posts were not applying environmental cleaning and disinfection for COVID-19 infection. One fourth of health extension workers were not using personal protective equipment's (Table 2).

**Table 2:** Infection prevention and control (IPC) measures by health posts in Ethiopia, 2021

Measures	Response options	
	Yes	No
Implemented any measures to create a COVID-19 safe environment (n= 343)	293(85.2)	51(14.8)
Screening of all patients and clients (n=193)	143(48.8)	150(51.2)
Distancing of at least 2 meters between patients and clients	268(91.5)	25(8.5)
Displaying instructions on hand, prevention and control measures of COVID-19 and respiratory hygiene practices for clients	241(82.3)	52(17.7)
Hand hygiene stations	265(90.4)	28(9.6)
Use of PPE by HEW	269(91.8)	24(8.2)
Environment cleaning and disinfection	154(52.6)	139(47.4)

**Figure 2:**
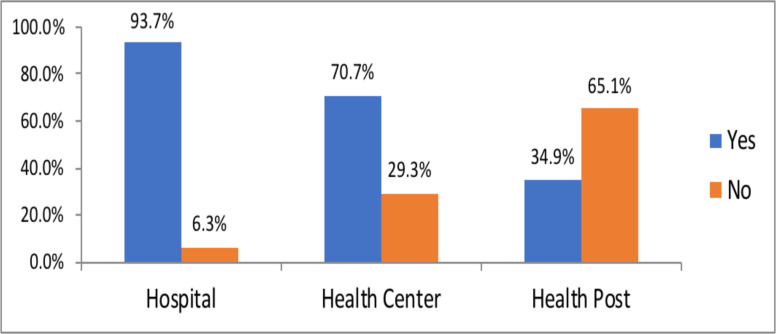
Availability of COVID-19 infection Prevention and Control Protocol at PHCUs in Ethiopia. 2021

**Availability of IPC guideline and protocol at PHCUs in Ethiopia:** Majority of health centers and primary hospitals have COVID -19 infection prevention and control protocols. In contrast to this, nearly seventy percent of health posts did not have COVID -19 IPC protocols (Figure 2).

**Availability of Personal Protective Equipment (PPE) at PHCUs in Ethiopia:** The presence of key COVID-19 prevention items indicates implementation of infection prevention and control. At the time of assessment, goggles were found to be the least available followed by respiratory mask whereas sanitizer was the most widely available. The overall average percentage of primary healthcare units with personal protective equipment was 50.07%. Dire-Dawa with highest (68.8%) followed by Oromia region with 59.8% (Table 3).

**Table 3:** percentage of PHCUs with COVID infection prevention items by region, Ethiopia, 2021

Region	Gown	Gloves	Respiratory Masks (N95 or FFP2)	Masks, medical/Surgical	Sanitizer	Soap for hand washing	Goggles
Amhara	57.6	84.8	9.1	81.8	80.3	50.0	4.5
Oromia	90.6	85.9	15.6	85.9	89.1	48.4	3.1
Sidama	38.1	81.0	11.9	73.8	57.1	40.5	2.4
SNNP	44.6	84.2	10.8	79.1	80.6	57.6	1.4
Dire-Dawa	66.7	93.9	42.4	93.9	90.9	51.5	42.4

**Management of Suspected and Confirmed COVID-19 Cases at PHCUs in Ethiopia:** Four Hospitals used both PCR and RDT tests and four hospitals were not doing any tests for COVID-19. Twenty-six health centers do RDT tests while about eighteen of them were not testing for COVID-19. More than two thirds of hospitals and health centers assigned focal person for COVID-19 service coordination. 68.8% of hospitals and 55.4% health centers have standard operation procedures for the management of patients with suspected or confirmed COVID-19 respectively. More than two third of hospitals and half of the health centers performed diagnostic tests for COVID-19 cases (Table 4).

**Table 4:** Management of suspected and confirmed COVID-19 cases at hospitals and health centers, in Ethiopia, 2021

Activities	Hospital (n =16)		Health Center (n= 92)	
	
	Yes n(%)	No n(%)	Yes n(%)	No n(%)
Focal person or team responsible for COVID-19 service coordination	14(87.5)	2(12.5)	79(85.9)	13(14.1)
Standard operation procedures for the management of patients with suspected or confirmed COVID-19	11(68.8)	5(31.2)	51(55.4)	41(44.6)
Collecting specimens from patients to diagnose COVID-19	14(87.5)	2(12.5)	42(45.6)	50(5.4)
Functional specimen transport system	10(62.5)	6(37.5)	57(61.9)	35(38.1)
Facility seen patients with suspected COVID-19 in the last 12 months	15(93.8)	1(6.2)	64(69.6)	28(30.4)
Checked for COVID-19 symptoms	16(100)	-	72(78.3)	20(21.7)
Measure O_2_ saturation with pulse oximeter	16(100)	-	39(42.4)	53(57.6.)
Referred the patient to specialized care	11(68.8)	5(31.2)	56(60.9)	36(39.1)
Performed diagnostic test	12(75)	4(25)	37(40.2)	55(59.8)

## Discussion

The study assessed the COVID-19 infection prevention preparedness, practices and its implementations in the primary health care units in Ethiopia. Preparedness of the primary health care units to prevent the transmission of COVID-19 is an immediate priority to safeguard patients and healthcare workers, protect risk groups, reduce the demand for specialized healthcare, and to minimize the spread of the pandemic to other healthcare facilities and the wider community (11).

Majority of PHCUs have designated IPC focal person and implemented measures to create a COVID -19 safe environment. There are gaps in on temperature screening, applying at least 2 meters patient/clients distancing, hand hygiene, screening and triage at all PHCUs. But the recommendation on Infection prevention and control (IPC) is to use appropriate PPE, proper hand washing, and hand hygiene were critical in preventing the transmission and risk of infection of COVID-19 in healthcare settings. The use of appropriate PPE by healthcare workers in particular during the current COVID-19 pandemic is highly recommended and the national and international safety protocols for healthcare workers should be strictly followed (12). Similarly, different studies recommend that all the pillars for infection prevention and control should be there to effectively halt chain of COVID-19 infections (13). When IPC systems are strengthened, multifocal responsible staff needs to assigned, and then responsive actions can be taken to address the identified needs (14).

This study revealed that there was shortage of personal protective equipment in hospitals, health centers and health posts with different magnitudes. Goggles were found to be the least available followed by respiratory mask whereas sanitizer was the most widely available. But different studies and world health organization recommends availing and using necessary PPE during all patient care and particularly strictly using PPE is mandatory during COVID-19 pandemic (15).

Twelve hospitals and thirty-seven health centers have capacity to diagnosis COVID-19 (PCR test, RDT test or both). But, four hospitals and fifty-five health centers did not have any of this diagnostic capacity. There are gaps on specimen transport system, following standard operational procedures in some hospitals and health cneters.

The current study covers a large number of health facilities from different settings at the national level and used WHO tools with expertise modification as a strength, there was no evidence to show a temporal relationship between the IPC readiness and practice during COVID-19 and factors that can act as enablers and barriers for IPC and PPE provision.

The study revealed that there are significant gaps on Infection prevention and control practice and readiness in term of assigning focal person for COVID-19 coordination, shortage of personal protective equipment, diagnosing COVID- 19, isolation and specimen transportation problem, lack of call centers. We recommend the regional health bureaus and ministry of health Ethiopia, and other supporting partners to work on feeling the gaps to solve the related infection transmission problem.
